# Differential gene expression of Asian citrus psyllids infected with ‘*Ca.* Liberibacter asiaticus’ reveals hyper-susceptibility to invasion by instar fourth-fifth and teneral adult stages

**DOI:** 10.3389/fpls.2023.1229620

**Published:** 2023-08-17

**Authors:** Ruifeng He, Tonja W. Fisher, Surya Saha, Kirsten Peiz-Stelinski, Mark A. Willis, David R. Gang, Judith K. Brown

**Affiliations:** ^1^Institute of Biological Chemistry, Washington State University, Pullman, WA, United States; ^2^Soybean Genomics and Improvement Laboratory, US Department of Agriculture (USDA)-Agricultural Research Service (ARS), Beltsville, MD, United States; ^3^School of Plant Sciences, University of Arizona, Tucson, AZ, United States; ^4^Sol Genomics Network, Boyce Thompson Institute, Ithaca, NY, United States; ^5^School of Animal and Comparative Biomedical Sciences, University of Arizona, Tucson, AZ, United States; ^6^Citrus Research and Education Center, Department of Entomology and Nematology, University of Florida, Lake Alfred, FL, United States

**Keywords:** circulative, *Diaphorina citri*, host-pathogen interactions, propagative transmission, psyllidae, RNA-seq analysis

## Abstract

The bacterial pathogen *Candidatus* Liberibacter asiaticus (CLas) is the causal agent of citrus greening disease. This unusual plant pathogenic bacterium also infects its psyllid host, the Asian citrus psyllid (ACP). To investigate gene expression profiles with a focus on genes involved in infection and circulation within the psyllid host of CLas, RNA-seq libraries were constructed from CLas-infected and CLas-free ACP representing the five different developmental stages, namely, nymphal instars 1-2, 3, and 4-5, and teneral and mature adults. The Gbp paired-end reads (296) representing the transcriptional landscape of ACP across all life stages and the official gene set (OGSv3) were annotated based on the chromosomal-length v3 reference genome and used for *de novo* transcript discovery resulting in 25,410 genes with 124,177 isoforms. Differential expression analysis across all ACP developmental stages revealed instar-specific responses to CLas infection, with greater overall responses by nymphal instars, compared to mature adults. More genes were over-or under-expressed in the 4-5^th^ nymphal instars and young (teneral) adults than in instars 1-3, or mature adults, indicating that late immature instars and young maturing adults were highly responsive to CLas infection. Genes identified with potential for direct or indirect involvement in the ACP-CLas circulative, propagative transmission pathway were predominantly responsive during early invasion and infection processes and included canonical cytoskeletal remodeling and endo-exocytosis pathway genes. Genes with predicted functions in defense, development, and immunity exhibited the greatest responsiveness to CLas infection. These results shed new light on ACP-CLas interactions essential for pathogenesis of the psyllid host, some that share striking similarities with effector protein-animal host mechanisms reported for other culturable and/or fastidious bacterial- or viral- host pathosystems.

## Introduction

The Asian citrus psyllid (ACP), *Diaphorina citri* is a phloem-feeding insect classified in the family, Liviidae (Order Hemiptera; Suborder Homoptera). It is the insect vector of the fastidious, plant phloem-inhabiting, gram-negative pathogen bacterium ‘*Candidatus* Liberibacter asiaticus’ (CLas), causal agent of the now widespread citrus greening disease that has affected the citrus industry, worldwide (Gottwald, 2010). The psyllid-CLas pathosystem has been problematic in other plant hosts in the subfamily, Aurantioideae, family Rutaceae (Grafton-Cardwell et al., 2013; Hall et al, 2013). In the United States, citrus greening has spread throughout all major citrus-growing areas in Florida and is responsible for outbreaks in commercial citrus in California, Texas, and other U.S. citrus-growing states (Stokstad, 2012). Invasions of *D. citri* have been reported in Arizona, but CLas has not been detected there in citrus trees. Since the initial discovery in Florida in 2005, and spread to other production areas in the U.S. the production of oranges has been reduced more than 50% (Ledford, 2017).

The ACP life cycle consists of seven stages, five nymphal instars (1~5), the teneral adult, and the adult. The average developmental time from egg to adult ranges from 14.1 days at 28°C to 49.3 days at 15°C (Hall et al, 2013). The mode of ACP-mediated psyllid transmission is persistent, and CLas is circulative and propagative in the vector. Psyllids ingests CLas while feeding on the phloem of infected plants, and CLas passes from the food canal into the alimentary canal (gut) where it multiplies and forms extensive biofilms (Wang and Trivedi, 2013). From the gut, CLas enters the hemolymph where it becomes motile, circulates to the oral region, and presumably enters the salivary glands. From the salivary glands, CLas cells are inoculated to the plant phloem in salivary contents during psyllid feeding (Ammar et al., 2011).

Extremely low-level transmission of CLas does from parent to offspring (transovarial) has been reported in laboratory studies, however, it may not be biologically relevant (Pelz-Stelinski et al., 2010; Kelley and Pelz-Stelinski, 2019). If viable in nature, transovarial transmission could feasibly provide a fail-safe mechanism to ensure CLas survival in a fraction of the ACP population (Vyas et al., 2015). A very low frequency of sexual transmission between males and females has been documented (Mann et al., 2011). Studies have shown rates of infection in ACP adults harboring detectable CLas, reared from the egg to adult stage on CLas-infected citrus plants, averaged ~40% and ranged from 30%-80% depending on the plant host growth-stage, season, temperature, humidity, and other environmental factors (Gottwald, 2010). No difference in inoculation efficiency has been reported for male and female psyllids (Ammar et al., 2020).

Young ACP nymphal stages acquire CLas and the adults transmit the bacterium and are involved in transmission and tree-to-tree spread (Kelley and Pelz-Stelinski, 2019; Ammar et al., 2020). The ACP nymphal stages acquire CLas at high rates that range from 60 to 100%. By comparison, even after a 5-week acquisition-access period (AAP) on infected plants, only 40% of the adults have acquired the pathogen (Pelz-Stelinski et al., 2010). Thus, CLas is efficiently transmitted by ACP adults only when acquired during the nymphal stage. Infection of adults and nymphal instars by CLas does not cause mortality. In adults, CLas infection has little discernable effect on longevity, and potential negative effects are offset by an increased fecundity (Pelz-Stelinski and Killiny, 2016; Ren et al., 2016). Although CLas multiplies in both the nymphs and adults, it accumulates to higher levels and in shorter periods of time by nymphs than by adults (Ammar El et al., 2016). The specific ACP nymphal stage or stages primarily involved in CLas ingestion that results in adult transmission have not been determined.

Liberibacter-encoded effector proteins have been posited as essential for infection of and systemic circulation within the ACP vector. After ingestion, CLas invades and colonizes the gut. After extensive multiplication, it utilizes exocytosis to exit, enter the hemolymph, and gain entry into the salivary glands where acquisition occurs (Cicero et al., 2017; Kruse et al., 2017). A number of psyllid proteins have been implicated in*’Ca*. Liberibacter’ effector interactions during ingestion, adhesion, multiplication, biofilm formation, circulation, and the acquisition phases (Fisher et al., 2014; Vyas et al., 2015). While differentially expressed CLas-responsive proteins have been identified in ACP nymphs and adults of ACP (Ramsey et al., 2017; Ramsey et al., 2022) and the related pathosystem involving the potato psyllid (PoP), *Bactericera cockerelli* and ‘*Ca*. Liberibacter solanacearum’ (CLso) (Vyas et al., 2015), the interactions between ACP and CLas throughout the developmental stages have not been explored in detail to enable characterization of age-related transcriptional dynamics. Previously, CLas-infected and uninfected nymphs and adult differential expression profiles have implicated developmentally regulated, stage-specific gene expression and/or proteins indicative mechanisms associate with gut invasion and salivary glands acquisition (Fisher et al., 2014; Vyas et al., 2015; Ramsey et al., 2017; Ramsey et al., 2022), and in addition for ACP, adult stage-specific transmission. Evidence of differential expression of defense-, stress-, and nutritionally related genes among the different ACP life history stages are hypothesized to reflect the distinct evolutionary history of ACP-CLas host-parasite interactions. Detailed analysis of the developmental physiology of CLas-infected psyllid and plant hosts could help explain the basis for the observed bimodal immature/adult acquisition/transmission and provide new insights in the unusual dual animal-plant host strategy of “*Ca*. Liberibacter” spp.

Exploiting RNA interference (RNAi) for managing insect pests and pathogens in agricultural crops is of increasing interest (Jain et al., 2021). RNA interference involves naturally occurring biochemical processes associated with organismal defenses that are highly conserved among eukaryotes. Triggering an RNAi response canonically results in gene silencing in which a foreign mRNA is targeted for degradation to impede gene expression or translation by sequence-specific, small interfering RNAs (siRNAs) complementary to the target mRNA (Hannon, 2002; Mito et al., 2011). In insects, exogenous double stranded RNAs (dsRNAs) have been used to induce RNAi pathway activation in a recipient insect to cause mortality or other phenotypes of interest. Silencing ACP genes to cause death or silence genes that impede efficient CLas transmission relies in part on functional genomic studies to gain a deeper understanding of basic ACP biology and of ACP-CLas interactions that facilitate multiplication, invasion, and systemic infection of the psyllid host and vector of this plant pathogen. The feasibility of RNAi in the potato psyllid (Mondal et al., 2022) and ACP has been demonstrated (El-Shesheny et al., 2013; Killiny et al., 2014; Kishk et al., 2017; Yu et al., 2017; Santos-Ortega and Killiny, 2018; Pacheco et al., 2020; Dos Santos Silva et al., 2022; Guo et al., 2022; Paredes-Montero et al., 2022) and functional genomics and proteomics studies have begun to inform effective and specific RNAi targets for PoP and ACP (Fisher et al., 2014; Vyas et al., 2015; Yang et al., 2022).

The objective of this study was to analyze sequence psyllid stage-specific transcriptomes and identify differentially expressed genes in ACP nymphal instars, 1-2, 3, and 4,5, and two developmental adult stages, the teneral stage, which are not yet reproductively mature, and reproductive, mature adults. The goal is to dissect instar-specific ACP responses to CLas invasion, multiplication, and systemic infection of the insect host. Of particular interest were differentially expressed genes (DEGs) predicted to contribute to the early-instar susceptibility to CLas gut invasion and early multiplication and to down-stream processes leading to systemic infection of the older immature (4-5) instars, the teneral (immature) adults. The rationale is that adults, which manifest transmission-competency, are so, only when CLas acquisition occurs during nymphal developmental stages when presumed barriers that impede adult acquisition do not exist and/or may be relaxed.

## Materials and methods

### Psyllid colonies

The CLas-infected and -uninfected ACP colonies were reared in laboratory cultures maintained on a CLas host (Citrus spp.) (CLas-infected) or a CLas-immune rutaceous plant species (CLas-free). Cultures were established in 2005 from a field population collected in Polk Co., FL (28.0’ N, 81.9’ W) prior to the establishment of HLB. Cultures were reared continuously and serially transferred periodically to the same host species, ‘Valencia’ sweet orange (*Citrus sinensis* (L.) plants, at the University of Florida Citrus Research and Education Center (maintained by coauthor Dr. K.S. Pelz-Stelinski, Lake Alfred, FL). The ACP colonies were maintained year-round on caged citrus trees, with ACP+ and ACP- colonies in separate walk-in growth rooms, IFAS Lake Alfred Research Center, FL for more than 10 years. The colonies have been assayed routinely for ACP presence (initially, seasonal CLas load), and in the CLas-free colony, for CLas absence. An established method, qPCR amplification of the CLas 16S rRNA gene, was used for CLas detection in psyllids (Li et al., 2006; Martini et al., 2015).

Five stages of nymphs (1–5 instar) and two stages of adults (teneral adult and mature adult) were collected, with 150 1^st^-3^rd^ instars and 100 4^th^-5^th^ instars (I-4,5), teneral adults (TA) and mature adults (MA) (also known as post teneral adults (PTA)) per sample, with 6 replicates for each. Males and females were collected in pairs to achieve nearly equal numbers of both, so that a comprehensive accurate representation of the transcriptome could be generated over the entire range of adult and nymph life stages. Live psyllids were collected from colonies and were frozen in liquid N_2_, lightly crushed in 0.5 ml Trizol (Invitrogen, Carlsbad, CA), and shipped overnight on dry ice to Washington State University where they were stored at -80°C until use.

### RNA isolation and quality control

For RNA extraction, 0.2 ml chloroform was added to 0.5 ml Trizol homogenate, followed by vigorous sample shaking for 30 s. Samples were allowed to rest for 3 min at room temperature, followed by centrifugation at 12,000 ×g for 15 min at 4°C to separate organic and aqueous phases. The aqueous phase (150-200 µL) was transferred to a sterile RNase-free tube and an equal volume of 100% EtOH was added, with mixing. The RNA was purified using the RNeasy Mini Kit (Qiagen, Valencia, CA), according to the manufacturer’s protocol. The Trizol RNA purification protocol that includes a DNase treatment step was implemented., according to the manufacturer’s instructions. The quality and quantity of the RNA samples was analyzed with a NanoDrop 2000 Spectrophotometer (Thermo Scientific, Wilmington, DE). The integrity of the RNA preparations was confirmed using an Agilent 2100 Bioanalyzer (Agilent Technologies Inc., Santa Clara, CA).

### Library construction and Illumina sequencing

The Illumina libraries were constructed from RNA transcripts purified using the TruSeq RNA Sample Preparation Kit v2, Cat. # RS-122-2002 (Set B) from Illumina. In brief, the poly(A) RNA was isolated from 2 μg of total RNA from each sample using magnetic oligo (dT) beads. Following purification, the mRNA was fragmented by zinc treatment at 94°C for 3 min and reverse-transcribed to synthesize first strand cDNA using SuperScript II reverse transcriptase (Invitrogen, Carlsbad, California) and random primers. Second-strand cDNA synthesis was performed, with the products then being subjected to end-repair and phosphorylation, and addition of an “A” base to the 3´ ends of the blunt phosphorylated DNA fragments. Illumina multiple indexing adapters were ligated to the fragments, as described by Illumina’s TruSeq RNA Sample Preparation V2 Guide (Illumina). The cDNA fragments flanked by Illumina PE adapters were selected and purified by AMPure XP beads for downstream enrichment. The cDNA fragments were amplified by PCR Primers PE 1.0 and PE 2.0 (Illumina) that anneal to the ends of the adapters, using the PCR program of 30 s at 98°C followed by 15 cycles of 10 s at 98°C, 30 s at 60°C, 30 s at 72°C and a final elongation step of 5 min at 72°C. The products were purified using AMPure XP beads to create an Illumina paired-end library. Library quality control was performed with a Bioanalyzer DNA 1000 Chip Series II (Agilent). A qPCR method was employed for quantifying libraries in advance of generating clusters. The libraries were diluted to a final concentration of 10 nM. The paired-end libraries were applied for cluster generation at a concentration of 10 pM on a flowcell in a cBOT (Illumina). Sequencing was performed on an Illumina HiSeq2500 platform by Macrogen with one lane for 12 pooled libraries (total 60 libraries in 5 lanes) to generate 2 × 150 bp paired end reads. The base-calling and quality value calculations were performed by the Illumina data processing pipeline CASAVA v1.8.4 and v1.7.0, respectively. Various quality controls including removal of reads containing primer/adaptor sequences, trimming read length and filtering high-quality reads based on the score values, were performed using Illumina CASAVA v1.7.0.

### Read mapping and identification of DEGs

The ACP reference genome Version 3 and official gene set version 3 (OGSv3) annotation files (Hosmani et al., 2019) were downloaded from the ACP genome website (https://www.citrusgreening.org/organism/Diaphorina_citri/genome). The raw sequencing reads were assessed using FastQC-v0.11.3 (Andrews) and trimmed for adapters and low quality using Trimmomatic-0.33 (Bolger et al., 2014). All cleaned reads were mapped to the ACP reference genome OGSv3 using HISAT2 (Kim et al., 2019). Mapped files were sorted with samtools rocksort. After sorting, a genome-guided transcriptome assembly described by Saha et al. (Saha et al., 2017) was performed using StringTie (Pertea et al., 2015). The reference genome and gene model annotation files were downloaded from the genome website (https://www.citrusgreening.org/organism/Diaphorina_citri/genome).

Differential expression analysis between the CLas-infected (infected) treatment and CLas-free (uninfected) control in the same stages (intra-stage) or the CLas-infected (infected) treatments between different stages (inter-stage) were performed using the DESeq2 R package. The False Discovery Rate (FDR) of 5% and *P*-value 0.05 were set as the threshold for significant differential expression. The hierarchical cluster analysis of differentially expressed genes (DEGs) was conducted using MeV (multi-experiment viewer) software. The significantly DEGs between the two arbitrary samples were identified based on the following thresholds: |log2 (fold-change (A/B))| > 1 and corrected *P*-value < 0.05. A and B represent the normalized expression of genes in any two samples, respectively.

### Annotation, Gene Ontology and KEGG enrichment analysis of DEGs

We supplied the OGSv3 annotation to StringTie during reference-guided transcript assembly so that it preserved the connection between the gene name in OGSv3 and the *de novo* assembled MSTRG transcripts reported by it. This information was used for extracting the functional annotations of the transcripts from OGSv3 genes, which were annotated using protein orthologs and the AHRD pipeline (Hosmani et al., 2019).

Ortholog assignment and pathway mapping were carried out via the KEGG (Kyoto Encyclopedia of Genes and Genomes) Automatic Annotation Server with the BBH (bi-directional best hit) method (http://www.genome.jp/kegg/kaas/) (Moriya et al., 2007; Kanehisa et al., 2016). The differentially expressed (fold-change ≥ 2 and p<0.05) genes (DEGs)/genes between uninfected and infected from different stages were mapped to KEGG biochemical pathways by BLAST comparisons against a set of orthologous groups in KEGG EGENES, resulting in the KEGG Orthology (KO) assignments and the Enzyme Commission (EC) distribution in the pathway databases. The following six data sets were submitted to KAAS; for simplicity in naming, the first letter indicates the ACP life stage, followed by a number(s) indicating larval (or nymphal) instar stage(s), with the last letter, being an ‘L’ (Liberibacter) if infected with CLas: 1) all of the ACP genes, 2) the genes differentially expressed (fold-change ≥ 2 and p<0.05) between CLas-free instars 1-2 (I12) and CLas-infected instars 1-2 (I12L) comparisons, 3) the genes differentially expressed (p<0.05) between CLas-free instar 3 (I3) and CLas-infected instar 3 (I3L) comparisons, 4) the genes differentially expressed (p<0.05) between CLas-free instars 4-5 (I45), and CLas-infected instars 4-5 (I45L) comparisons, 5) the genes differentially expressed (p<0.05) between CLas-free teneral adult (TA) and CLas-infected teneral adult (TAL) comparisons, and 6) the genes differentially expressed (p<0.05) between CLas-free mature adult (MA) and CLas-infected mature adult (MAL) comparisons. All of the results were obtained using the TCW query and display interface, hence, are reproducible, with one exception. The KEGG results are provided in an additional file as an Appendix that explains how each table and figure was generated.

### Illumina data

The Illumina paired end transcript reads from the five stages of nymphs (1–5 instar) and two stages of adults (teneral adult and mature adult) have been uploaded to NCBI SRA under Bioproject PRJNA746599 (https://www.ncbi.nlm.nih.gov/bioproject/?term=PRJNA746599) with accessions: SRX20741680 ~ SRX20741739. To improve the accessibility and usability of this data for the citrus greening research community, data will be incorporated in future versions of the Psyllid Expression Network (https://cgen.citrusgreening.org/). This visualization tool is available to access publicly available transcriptomics data for *D.citri* from multiple tissues, life stages, infection states, and of the citrus host plant, in an expression cube for comparative analyses.

## Results and discussion

### *De novo* transcript discovery using a comprehensive sampling of expression data from every ACP life stage

Polyadenylated ACP RNA was isolated and used to construct 60 Illumina paired-end sequencing libraries, from pools of five developmental stages (instar 1-2, instar 3, instar 4-5, teneral adults, and mature adults) of CLas-free and CLas-infected ACP with six replicates each. Sequencing was carried out as described in the *Methods* section and producing 1,960 million paired-end 150 bp sequencing reads consisting of 296 G bp (**Additional file 1**).

**Figure 1:**
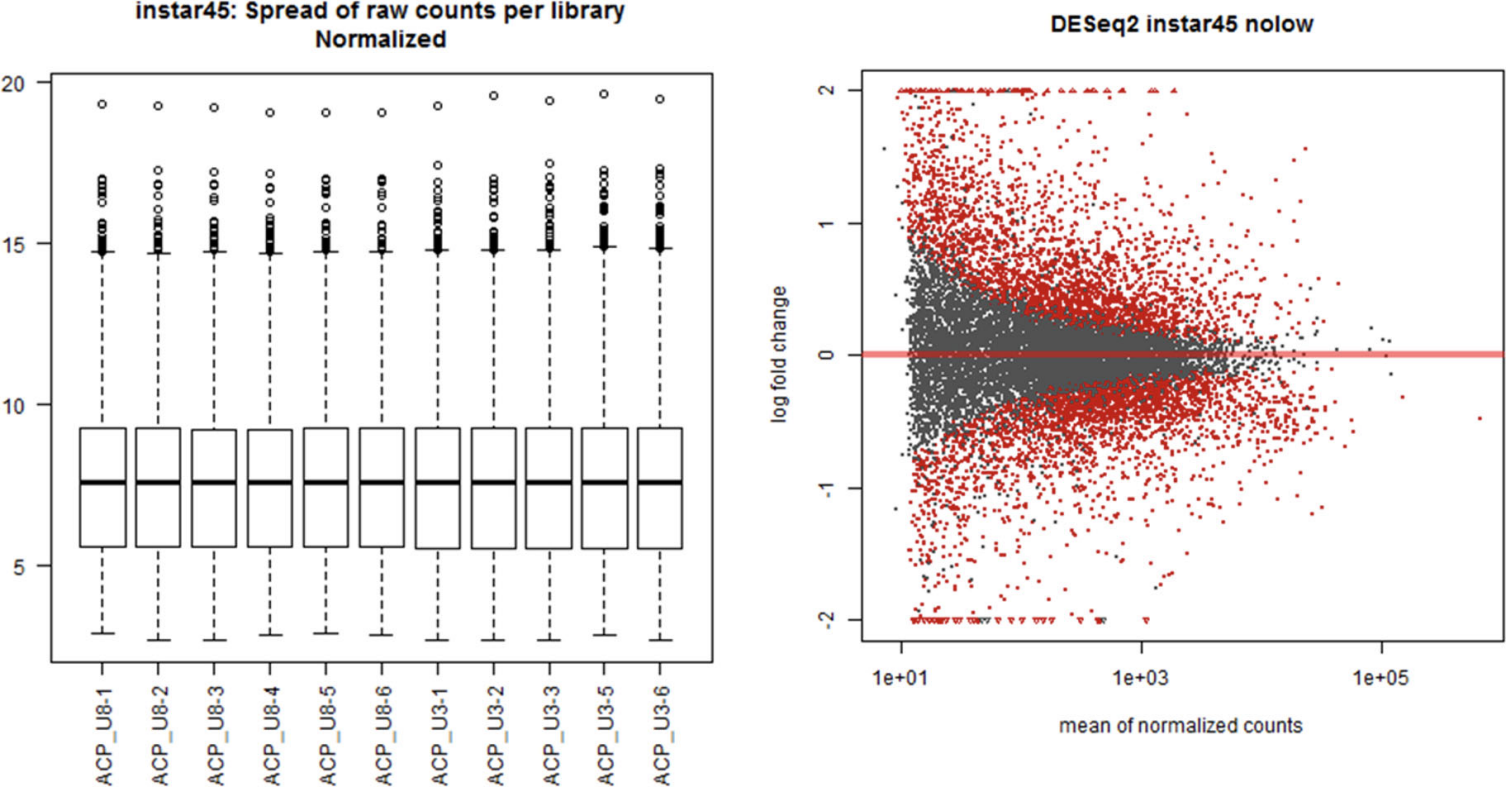
Sequencing data distribution and normalization for the Asian citrus psyllid (ACP) immature group consisting of 4^th^ and 5^th^ instars (I4-5).

**Figure 2:**
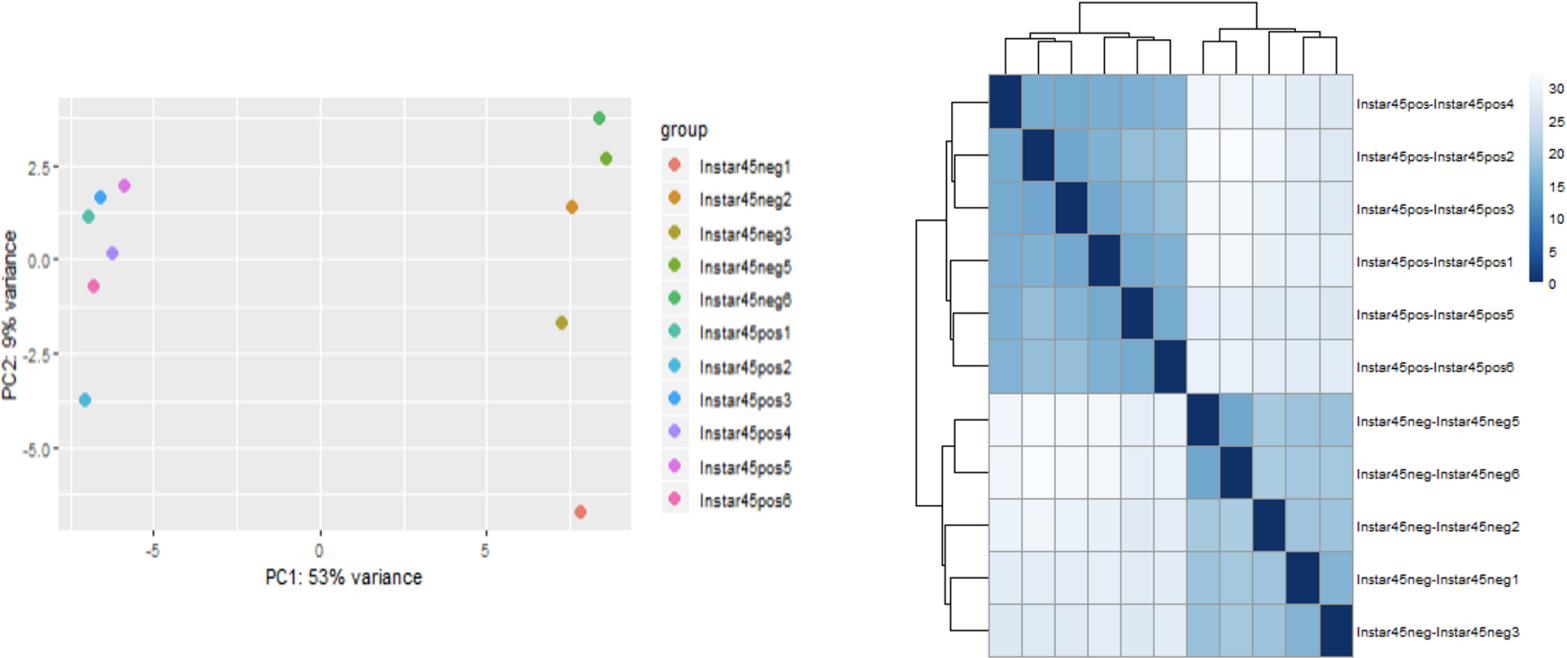
Principal component and pairwise correlation analysis of sequencing data for Asian citrus psyllid (ACP) immature group consisting of 4^th^ and 5^th^ instars (I4-5), infected or uninfected with ‘*Candidatus* Liberibacter asiaticus’.

Quality filtering removed approximately 2% low-quality reads found to be present in most of the replicated samples. Most replicates contained 4-7% rRNA and 5-12% mitochondrial RNA reads, which were also removed from downstream analysis. Principal Component Analysis (PCA) was carried out to determine if the sequencing data were representative and of high quality. The evaluation of sequence data and PCA were carried out for all developmental stages/groups. However, the instar 4-5 (I45) group showed the most significant overall differential expression; it was selected and highlighted here to visually represent the overall trend (**Figures 1**, **2**). Importantly, the instar 4-5 (I45) group exhibited the most responsive stage to CLas infection/invasion, based on the extensive number of differentially expressed genes, compared to the other stages/groups, making it the most representative. In addition, one sample (ACP_U3-4 in **Figure 1** or Instar4,5 neg4 in **Figure 2**) failed to pass quality control and so the data were not included. An optimized index was built for the chromosomal length version 3.0 reference assembly with splice junctions based on official gene set (OGSv3) and overall, the alignments were found to be consistent at approximately 85-87%. The transcriptome data were used to extend the OGSv3 beta annotation, which resulted in the *de novo* transcript discovery of a total of 25,410 genes with 124,177 isoforms.

### Greater differential gene expression in instars 4-5 and more over-expressed genes compared to under-expressed expression in teneral adults

Among the total of assembled/aligned genes in each stage, 21805 genes were identified as significantly differentially expressed genes in response to CLas infection (with False Discovery Rate (FDR) of 5% and *P*-value < 0.05), including 4768 genes (33%) from instar 1-2 (I-1,2), 4901 genes (35%) from instar 3 (I-3), 6389 genes (44%) from instar 4-5 (I-4,5), 4843 genes (33%) from teneral adult **(**TA) and 904 genes (6%) from mature adult (MA) (**Table 1**). In general, there were more over-expressed than under-expressed genes (in total, 11032 over-expressed genes compared to 10773 under-expressed genes among different stages in response to CLas infection) (**Table 1**). These results showed more differentially expressed genes were associated with the nymphal instars (including instar 1-2, instar 3 and instar 4-5) compared to adults, indicating that instars are more responsive to CLas infection. In particular, the instar 4-5 stage contained more DEGs (3147 over-expressed and 3242 under-expressed, totaling 6389 genes), indicating instar 4-5 was much more sensitive/responsive than the others to CLas infection. Further, more over-expressed genes among the instar 4-5 and teneral adults than in other stages, with 3147 and 2639 genes over-expressed in immature instars 4-5 and teneral adults, respectively (**Table 1**).

**Table 1:** Summary of intra-stage differentially expressed genes in response to ‘Candidatus Liberibacter asiaticus’ (CLas) infection of ACP.

Intra-stages (Clas-infected vs. –uninfected ACP)	Up (%)	Down (%)	Total (%)
Instar 1-2 group	2320 (16%)	2448 (17%)	4768 (33%)
Instar 3	2409 (17%)	2492 (18%)	4901 (35%)
Instar 4-5 group	3147 (22%)	3242 (22%)	6389 (44%)
Teneral adult (TA)	2649 (18%)	2194 (15%)	4843 (33%)
Mature adult (MA)	507 (3.4%)	397 (2.6%)	904 (6%)
Total	11032	10773	21805

Differential gene expression in response to CLas infection was also compared among different stages (inter-stages). In total, 2314 DEGs were identified in nymphal instar 4-5 compared to instar 3, including 1248 over-expressed genes, which was much higher than other comparisons (**Table 2**). Nymphal instars 4-5 and teneral adults showed the largest changes in gene expression in response to CLas infection. Furthermore, there were more over-expressed genes (566 genes) than under-expressed genes (244 genes) in the teneral stage as compared with instar 4-5 (**Table 2**), indicating that CLas infection mainly causes up-regulation of ACP genes.

**Table 2:** Inter-stage differentially expressed genes in response to ‘*Candidatus* Liberibacter asiaticus’ (CLas) infection of ACP.

Inter-stages (Clas-infected ACP)	Up	Down	Total
Instar 3 vs. instar 1-2 group	584	545	1129
Instar 4-5 group vs. instar 3	1248	1066	2314
Teneral adult (TA) vs. instar 4-5 group	566	244	810
Mature adult (MA) vs. teneral adult (TA)	6	10	16
Total	2404	1865	4269

The differentially expressed genes identified herein were classified by predicted biological functions using KEGG analysis to obtain an overview of the processes altered by gene differential expression in response to CLas infection. Using KAAS (KEGG Automatic Annotation Server), which provides functional annotation of genes by BLAST comparisons against the manually curated KEGG GENES database to produce KO (KEGG Orthology) assignments and automatically generated KEGG pathways, the genes differentially expressed (FDR of 5% and *P*-value < 0.05) in the different life stages/groups, in response to CLas infection, were assigned to KEGG biochemical pathways.

**Figure 3:**
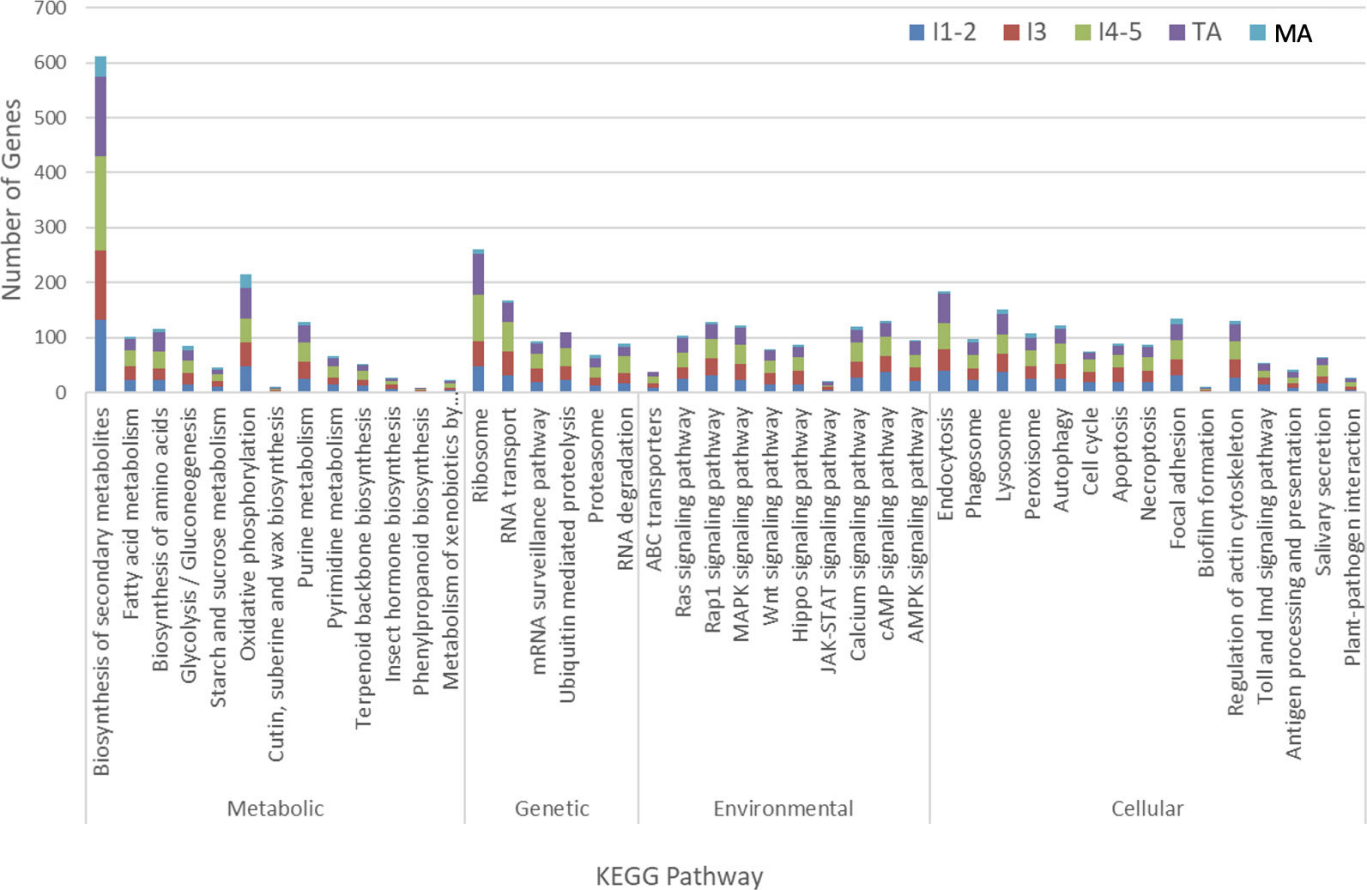
KEGG analysis of differentially expressed Asian citrus psyllid genes in response to ‘*Candidatus* Liberibacter asiaticus’ infection.

The genes assigned to nymphal instar 3, instars 4-5 and teneral adults dominated the pathways (**Figure 3**), with most being distributed in categories “biosynthesis of secondary metabolites” (127 gens from instar 3, 170 genes from instar 4-5 and 146 genes from teneral adults), “ribosome” (45 genes from instar 3, 84 genes from instar 4-5 and 76 genes from teneral adults), “oxidative phosphorylation” (43 genes from instar 3, 42 genes from instar 4-5 and 57 genes from teneral adults), and “endocytosis” (39 genes from instar 3, 49 genes from instar 4-5 and 53 genes from teneral adults), documenting significant transcriptional changes among the different ACP life stages in response to CLas infection (**Figure 3**). These observations also suggested that CLas infection resulted in more significant effects on the nymphal instars 4-5 and teneral adults, than to the three youngest immature instars (1,2 and 3) and mature adults.

### Unique and shared differentially expressed responsive genes between developmental stages

**Figure 4:**
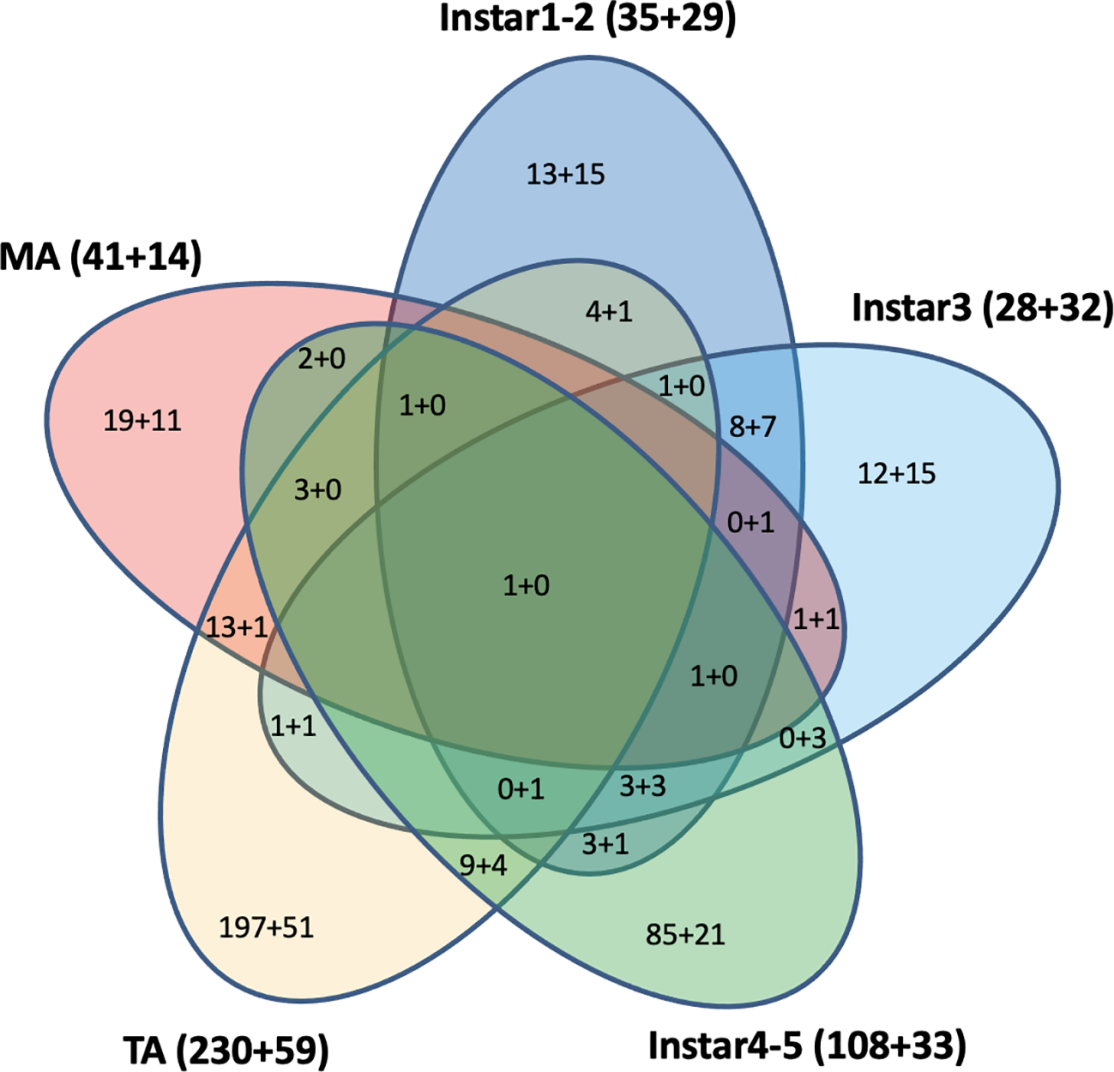
Differentially expressed KEGG pathway Asian citrus psyllid genes: up-regulated (indicated, as before ‘+’), compared to down-regulated (indicated, after ‘+’). Fold-changes >2, P<0.05.

Cross-development stage comparisons of CLas-responsive genes were performed to determine if bacterium infection had a universal effect on gene function and gene expression levels. Among these DEGs, some were shared by two, three, four, or five stages (**Figure 4**). For example, a U6 snRNA-associated Sm-like protein gene (MSTRG.13654) was highly over-expressed (more than 2-9-fold in different stages) and shared by all five life stages after CLas infection. This gene is involved in RNA processing and associated with RNA degradation and the spliceosome. In contrast, the H+/sugar cotransporter gene (MSTRG.17346) was under-expressed, a pattern that was shared in common by all four CLas-infected life stage groups examined here, except for the mature adult stage. Among the 609 DEGs with 2-fold differences that were assigned to KEGG pathways, 290 (48%) DEGs were from teneral adults and 140 (23%) DEGs were from instar 4-5, and approximately 64 (10%), 60 (10%), and 55 (9%) DEGs were from instar 1-2, instar 3 and mature adult respectively. Among these, 442 (73%) genes were over-expressed, whereas 167 (27%) genes were under-expressed. Among 290 DEGs from teneral adults and 140 DEGs from instar 4-5, 231 (80%) and 107 (76%) were over-expressed, respectively, whereas among the 60 DEGs from instar 3, 32 (53%) were under-expressed (**Figure 4**).

The 50 top over-expressed and 50 top under-expressed genes in response to CLas infection in most life stages are shown, highlighted (**Additional file 2** and **3**). Interestingly, the top 50 over-expressed genes were primarily associated with the teneral adult stage (41/50), while the top 50 under-expressed genes were distributed across different stages, however, the majority were associated with the teneral adult stage. The predominantly over-expressed genes were assigned to sugar metabolism and glycan degradation, signaling pathway, oxidative phosphorylation, ribosome, ubiquitin and collagen pathways. Among these, a calmodulin gene (MSTRG.16314) was overexpressed in three CLas-infected groups: instars 4-5, teneral adults and mature adults), with greater than 60-fold in the teneral adult stage, alone (**Additional file 2**). Calmodulin (CaM) is a multifunctional intermediate calcium-binding messenger protein and directly or indirectly has a functional role in nearly every physiological process. Calmodulin is involved in the activation of phosphorylase kinase that leads to cleavage of glucose from glycogen-by-glycogen phosphorylase (Nishizawa et al., 1988). Also, a number of sugar metabolism- and glycan degradation-related genes were over-expressed in response to CLas infection, strongly implicating pathogen modulation of primary energy-producing molecules. For example, a gene (MSTRG.18384) coding arylsulfatase B was over-expressed in all five stages, at greater than 7-fold in the instar 4-5 stage. This gene is involved in glycosaminoglycan degradation. In contrast, the most under-expressed genes were assigned to cytoskeleton proteins, membrane trafficking and exocytosis, peptidase inhibitors, and biofilm formation categories, suggestive of cytoskeletal remodeling to aid CLas invasion and multiplication, while potentially moderating the advancement of systemic invasion.

### Energy metabolism is altered in ACP in response to CLas infection

Many of the top 50 up- and under-expressed genes were mitochondrial and energy metabolism-related genes/proteins. For example, mitochondrial mRNA pseudo uridine synthase TRUB2 (MSTRG.9981) was over-expressed in stages instar 4-5 (I-4,5) and teneral adult (TA) (> 6-fold). In addition, an EMRE (essential MCU regulator, mitochondrial) gene (MSTRG.15478) and a ubiquinol-cytochrome c reductase subunit 9 (MSTRG.22133), which is involved in oxidative phosphorylation and energy metabolism, were also highly over-expressed in the teneral adult stage (TA) (**Additional file 2**). Also, an ATPase subunit b, mitochondrial (MSTRG.5785), involved in oxidative phosphorylation and energy metabolism, was under-expressed more than 4-fold after CLas infection in stage I45 compared to stage I3. Two ATP synthase genes (MSTRG.23375 and MSTRG.11595) were significantly over-expressed in stage TA (**Additional file 5**). This increase in the availability of ATP for expensive-energy driven processes is consistent with a previous report (Killiny et al., 2017) reported that the ATP levels were significantly higher in CLas-infected than in CLas-free psyllids. Gene expression analysis showed upregulation of ATP synthase subunits, while ATPase enzyme activity was lower in CLas-infected psyllids. Further, ATP synthase subunit expression was about 5-fold greater in CLas-infected ACP indicating that the CLas alters the energy metabolism of its insect vector. These results suggest such changes are not due solely to changes at the biochemical/metabolic level but rather that they are regulated by specific changes in expression of the genes that control those metabolic processes.

This latter result is consistent with the results of a recent study in which a total of 196 mitochondrial proteins were identified in the ACP gut proteome, and 25 of 26 differentially expressed proteins in CLas-infected ACP guts were downregulated, resulting in widespread depression of mitochondrial function (Kruse et al., 2017). In this study, however, the latter genes, analyzed for whole psyllids (not gut, only) were mostly over-expressed in senior nymphal stages (especially in instar 4-5) and adult stages (especially in TA), and under-expressed in junior nymphal stages, especially instar 1-2 and instar 3. These observations suggested that CLas infection regulates the ACP energy metabolism of the psyllid host and vector, most likely obtaining direct energy for multiplication and growth in the form of ATP from its host. This observation may further provide insights into requirements for CLas culturing *in vivo*. A recent transcriptome profiling of CLas in citrus and psyllids has also identified genes related to transcription or translation associated with resilience to plant host defense response that were upregulated in citrus. Strikingly, genes involved in energy generation were expressed at higher levels in ACP host, compared to the citrus host plant (De Francesco et al., 2022).

### Ubiquitination, cellular stress and apoptosis to CLas infection

Ubiquitin modification or ubiquitination has critical functions in recognition and clearance of certain invading bacteria through autophagy and also exert multiple effects on the host immune system. The ubiquitin-proteasome system (UPS) is a key signaling pathway in host responsiveness to bacterial or viral pathogen infection (Kocaturk and Gozuacik, 2018), and to infect their host, some bacterial effectors interfere with the process, for example, by catalyzing attachment of ubiquitin to host proteins and subvert cell functions (Dupont et al., 2010; Vozandychova et al., 2021)Ubiquitination of proteins regulates protein stability, receptor internalization, enzyme activity, and protein-protein interactions, including receptor-mediated endocytosis, signaling, and membrane protein trafficking (Raiborg and Stenmark, 2009). In this study, a number of ubiquitination- and proteasome-related proteins/genes were identified with predicted involvement in post-translational attachment of ubiquitin to a target proteins that function in cell cycle progression, and cell proliferation, and development. Most of these genes were over-expressed in CLas infected ACP, most notably in immature stages 4 and 5 (I-4,5) and teneral adults (TA) (**Additional file 7**), and further, some were represented among the top 50 DEGs (**Additional file 4**).

Interestingly, nearly all top over-expressed genes with greater than 1000-fold that were overexpressed in infected immature instars 4 and 5 (I-4,5), compared to infected immature instar 3 (I-3), were assigned to the ubiquitin-proteasome system (UPS) and membrane trafficking pathways, including SPRY domain-containing SOCS box protein 3 (MSTRG.18581), AN1-type zinc finger and ubiquitin domain-containing protein 1 (MSTRG.15367), dynein light chain roadblock-type (MSTRG.10226), 20S proteasome subunit beta 1 (MSTRG.6586), E3 ubiquitin-protein ligase RNF115/126 (MSTRG.16443). These proteins/genes are involved in bacterial infection, cell stress response, and the host immunological processing of endogenous antigens. These results indicated that the stage 4-5 instar (I-4,5) is a highly important stage or point of switching from somewhat benign interactions with the host, to accelerated CLas invasion, requiring a more robust response to CLas infection, based on the evidence that more DEGs dominated in the instar 4-5 stage. This is consistent with the previous identification of a number of ubiquitination and proteasome-related DEGs in the ACP midgut, in response to CLas infection (Yu et al., 2020) and supports the hypothesis that CLas may activate host ubiquitination to eliminate immune-related proteins.

Heat shock proteins (HSPs) are ubiquitous and conserved chaperones with cytoprotective activities that are known to be particularly prominent under pathological conditions through the initiation of protein folding, repair, and refolding to minimize cellular damage and apoptosis (Ikwegbue et al., 2017). The HSPs have been recognized as important immune-response proteins that combat biological and environmental stresses in well-studied insect systems (Aguilar et al., 2005; Gotz et al., 2012). Consistent with the recent report (Liu et al., 2020), in this study, several heat shock protein genes were among the top 50 under-expressed gene list, with some shared among the different life stages/groups (**Additional file 3**). This differential regulation of HSPs strongly suggests that CLas infection activates and then depresses the ACP immune system to modulate attack by the host at particular times in the infection process over others, relative to key ‘pathogenesis’ requirements required during each different host life stage or group (herein).

**Figure 5:**
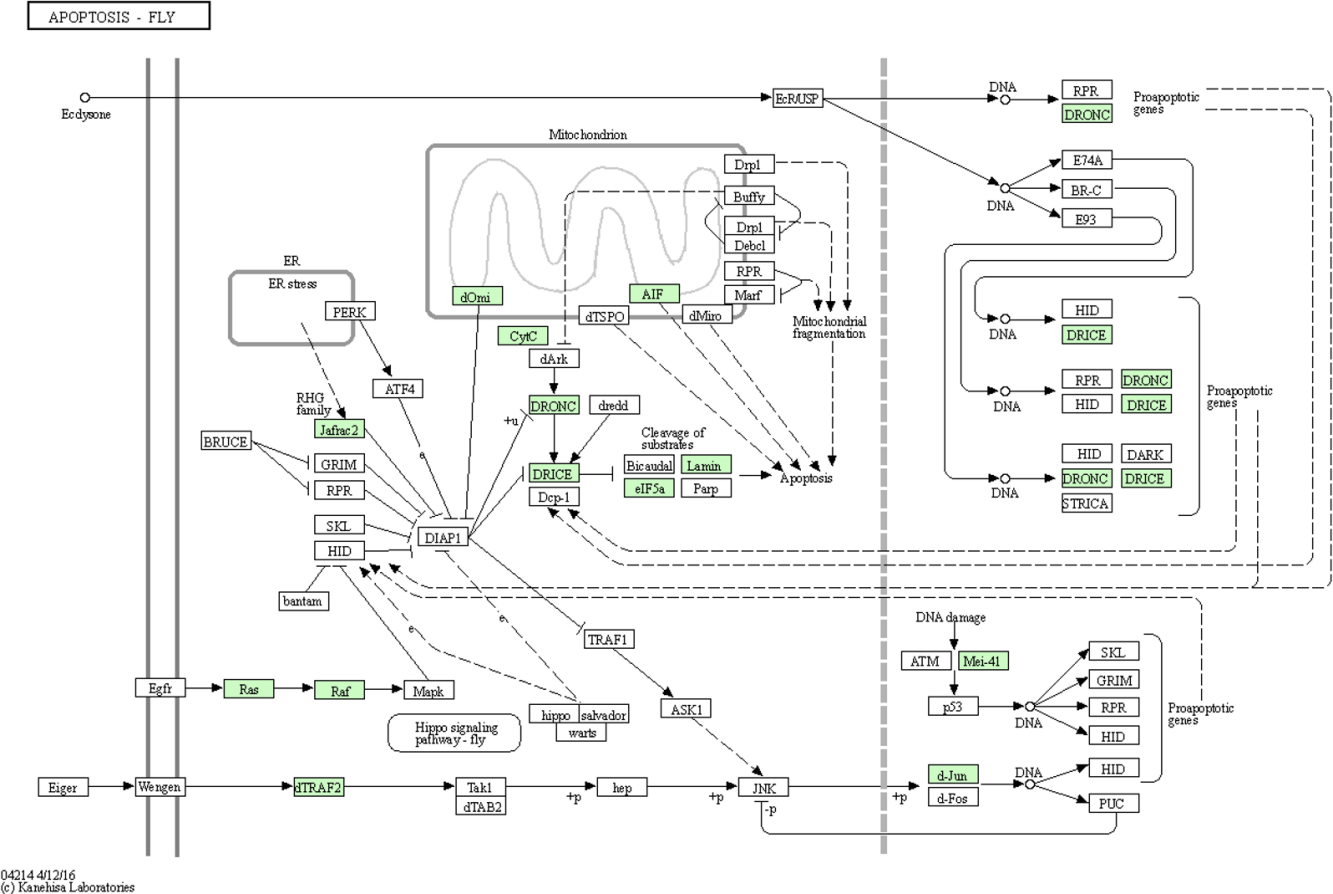
Apoptosis pathway diagram showing the 19 up-regulated genes (P<0.05) in the teneral adult stage, responsive to ‘*Candidatus* Liberibacter asiaticus’ infection. Assigned genes are shaded in green.

Apoptosis is a kind of programmed cell death that is important for many processes, among which are immunity, development, and cell homeostasis. Virus-associated apoptosis has been reported in the brown planthopper, *Nilaparvata lugens* (Stål), the vector of *Rice ragged stunt virus* (RRSV), in which RRSV is propagative. The RRSV was shown to induce apoptosis in the salivary gland cells of *N. lugens*, which was regulated in a caspase-dependent manner. The inhibition of expression of *N. lugens caspase-1* genes was found to significantly interfer with virus transmission (Huang et al., 2015). A recent study has reported apoptosis occurs in CLas-exposed ACP guts (Kruse et al., 2017). Significantly more nuclear fragmentation was observed in midgut cells of adults compared to ones of nymph, and protein biomarkers of apoptosis were more abundant in midguts of CLas(+) compared to infected adult insects (Ghanim et al., 2016; Kruse et al., 2017; Mann et al., 2018). Differences in histone cross-links observed between CLas(+) nymphs and adult *D. citri* suggested that while nuclear fragmentation and apoptosis were observed in adult midguts during CLas acquisition, nymph midgut nuclear morphology was largely unaffected by insect exposure to CLas (Ramsey et al., 2022). In this study, of the 90 ACP DEGs identified in this pathway, 20, 25, 24, 17, and 4 were significantly (p<0.05) differentially expressed in response to CLas infection of immature instar1-2, instar3, instar4-5, TA and MA stages, respectively (**Additional file 8**). The 19 up-regulated genes (p<0.05) in stage TA found to be most responsive to CLas infection were expressed in the apoptosis pathway diagram (**Figure 5**).

### Adhesion, endocytosis, and cytoskeleton changes potentially involved in entry and systemic invasion

Adhesion molecules are sticky cell-surface molecules that facilitate intercellular binding and communication and govern cell-to-cell interactions necessary for pathogen detection by the host. The plant-pathogenic bacterium *Xylella fastidiosa* adhesins binds to carbohydrates, an interaction essential for the initial cell attachment to the leafhopper vector, the requisite for bacterial transmission to the host plant (Killiny et al., 2012). Among the top-50 over-expressed gene list, a cell adhesion molecule related to protocadherin-15 (MSTRG.4446) was identified in the teneral adult stage. Interestingly, two under-expressed cell adhesion molecule genes (MSTRG.21105, MSTRG.19362), also among the top-50-list of genes, were identified in the youngest two immature instar stages (I12) and in mature ACP adult stage, respectively. Among DEGs, 134 genes assigned to the focal adhesion category were significantly (p < 0.05), differentially expressed in all stages, with more over-expressed (n=99) than under-expressed (n=35) genes (**Figure 3** and **Additional file 8**). Most of the over-expressed genes were assigned to cuticle proteins, serine/threonine-protein phosphatase, zinc finger protein, adhesive plaque matrix protein, Ras-related C3 botulinum toxin substrate 1, laminin, talin and vitellogenin, potentially indicative of adhesion leading to active cytoskeletal and endosomal remodeling, and/or endo-exocytosis in the insect vector gut, potentiating hemolymph entry and translocation (systemic spread) and/or salivary glands association and entry.

**Figure 6:**
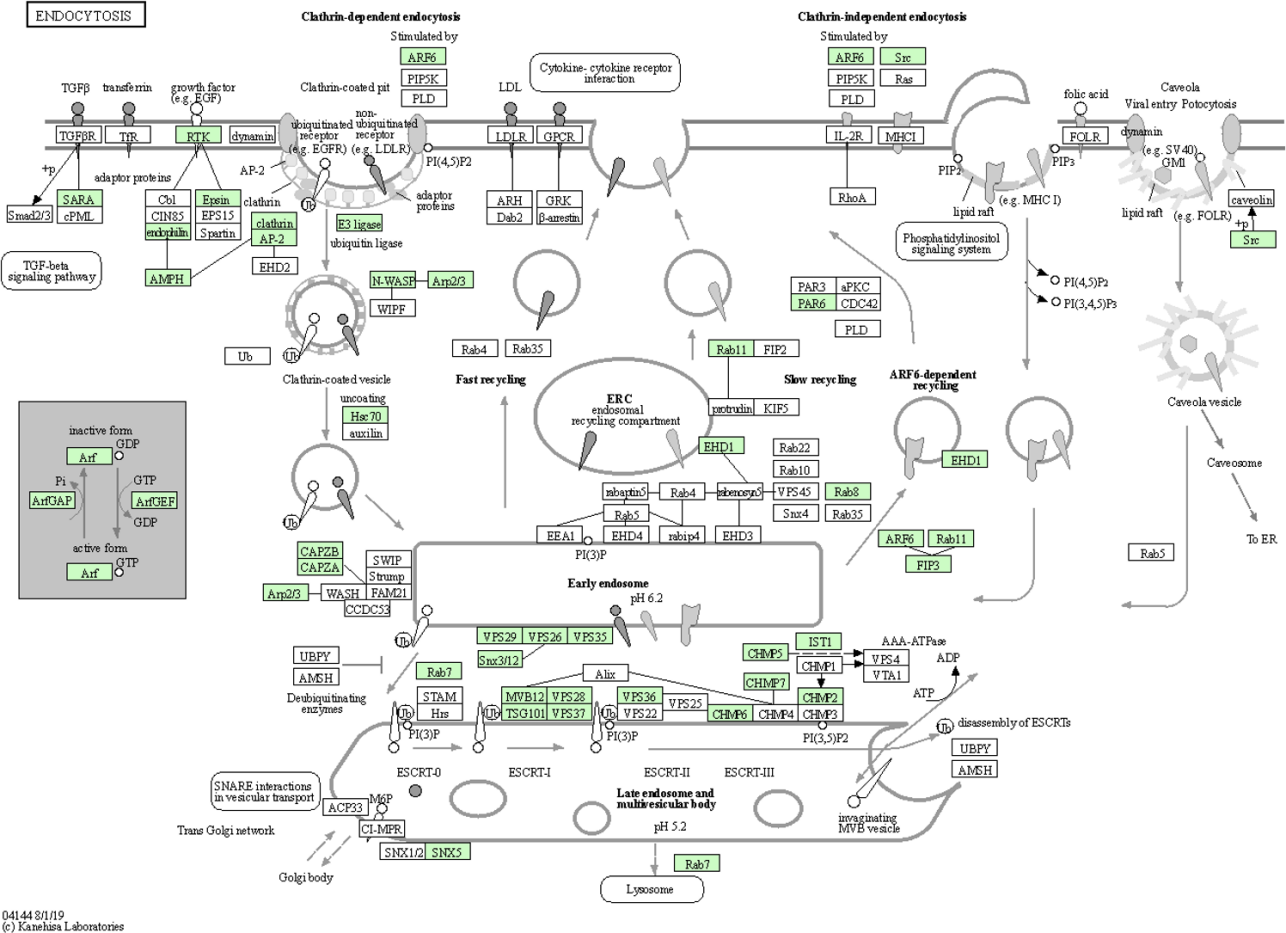
Endocytosis pathway diagram showing the 47 up-regulated genes (P<0.05) in the teneral adult stage, responsive to ‘*Candidatus* Liberibacter asiaticus’ infection. Assigned genes are shaded in green.

Endocytosis is known to facilitate host invasion by many viral and bacterial pathogens (Ungewickell and Hinrichsen, 2007; Glebov, 2020). More recently, evidence suggests that the severe acute respiratory syndrome coronavirus 2 (SARS-CoV-2) may employ distinct endocytic pathways for entry of the upper and lower respiratory tract, leading to the consideration of clinically approved drugs as potential candidates for repurposing them as blockers of the different potential routes for SARS-CoV-2 endocytosis (Glebov, 2020). The pathogen *Salmonella enterica* remodels the host cell endosomal system for efficient intravacuolar nutrition (Liss et al., 2017). Both biological adhesion and endocytosis appeared to be extremely important to CLas-ACP interactions, specifically, CLas-cell attachment during entry and later, for biofilm formation, and to facilitate exit from the gut to the lumen. Among 185 identified DEGs in the endocytosis pathway, they are distributed in different stages with more over-expressed in teneral adult stage (TA) (46 up- and 7 under-expressed), with a greater number being under-expressed in immature instars 4 and 5 (I4-5) (19 over-expressed and 30 under-expressed) in response to CLas infection (**Additional file 8**). The 47 genes that were most significantly up-regulated (p<0.05) in the TA stage, in response to CLas infection, were expressed in the endocytosis pathway diagram (**Figure 6**). Based on these results, it can be posited that the early ACP developmental stages, e.g. instars 1-3, were not as responsive to CLas, as are the late immature stages, instars 4-5, and teneral adults (TA), which is consistent with the results of a previous study (Vyas et al., 2015). Thus, the instars 4-5 exhibited the most actively-responsive stage to CLas infection/invasion processes underway, with many more genes showing differential expression in this stage than in the three (youngest) immature stages (I-1,2 and I-3), the latter stages when CLas is hypothesized to be targeted effectively by ACP host defenses (Fisher et al., 2014; Vyas et al., 2015), compared to the most being under-expressed genes in the two oldest immature instar stages (I-4,5). In the latter two stages (I-4,5), it may be that CLas down-regulates endocytosis-related gene expression to prevent death of ACP before CLas can be transmitted to the plant host.

The cytoskeleton is well-known for its roles in cell division, shape, cell motility and intracellular transport and has important functions in innate immunity and cellular self-defense against bacterial and viral pathogens (Mostowy and Shenoy, 2015). Characteristic of the arms race, bacterial pathogens have mechanisms to avoid or exploit the autophagy machinery (which utilizes the cytoskeleton) for intracellular survival (Mostowy, 2014). A recent quantitative analysis of ubiquitylome-proteome crosstalk showed that cytoskeleton proteins were associated with CLas infection of ACP, in that 21 lysine ubiquitinated proteins were associated with the cytoskeleton functions (Zhang et al., 2023). Further, the structure of the cytoskeleton has been shown to undergo modification in the *D. citri* midgut post-CLas infection (Ghanim et al., 2016), an observation that is supported by the previous *in silico* predictions based on comparative transcriptome analysis (Fisher et al., 2014; Vyas et al., 2015). Also, quantitative isotope-labeled cross-linker proteomics investigation revealed developmental variation in protein interactions and post-translational modifications in CLas infection of ACP. The most represented protein category among cross-linking proteins abundant in both ACP nymphs and adults were cytoskeleton/muscle protein complexes which might be expected to represent hallmark morphological and behavioral differences between different ACP life stages, reported in a previous study (Ramsey et al., 2022). These collective observations suggest that CLas differentially alters certain cytoskeletal proteins during invasion and infection processes as CLas pathogen infection of ACP proceeds.

In this study, 130 DEGs associated with regulation of the actin cytoskeleton were identified, and most were upregulated in the CLas-infected groups, compared to the CLas-free groups (**Additional file 8**). More importantly, some cytoskeleton-related genes (such as actin-binding protein gene MSTRG.12781, tubulin monoglycylase gene MSTRG.9444, katanin p60 ATPase-containing subunit A1 gene MSTRG.20422) were listed in the top-50 DEGs and were highly over-expressed in stages I45 and TA compared to stage I3 in response to CLas infection (**Additional file 4**). These results indicate that ACP responds strongly to CLas infection at the latter stages of nymphal development in a manner that appears to abate further infection/multiplication of the CLas pathogen, potentially explaining, at least in part, why ACP appears to be much more susceptible to long-term, successful colonization by CLas when infection occurs during early nymphal development stages, e.g. prior to instars 4,5 (I-4,5).

### Immune and defense responses

A number of immunity related genes (Kruse et al., 2017) responded at different stages of ACP development to CLas infection, including immune regulating signaling pathways, Toll signaling pathway, JAK/STAT pathway, and ABC transporter activity (**Table 3**). The Toll signaling pathway plays a key role in the innate immune system. This immune pathway is responsible for activation of antimicrobial peptides involved in the inhibition of dengue virus proliferation in symbiotic *Wolbachia*-infected mosquitoes (Pan et al., 2012). The Janus kinase/signal transducer of activators of transcription (JAK/STAT) pathway is linked to many developmental processes, in addition to a major role in immunity. A number of differentially expressed genes in these pathways were identified with more over-expressed than under-expressed genes (on average, 11% up- compared to 2% under-expressed) (**Table 3**). Among 244 genes assigned to the antimicrobial response category, 48 (20%) were over-expressed, and 10 (4%) were under-expressed, including, notably, a transferrin gene exhibiting 9-fold and 3-fold upregulation in instars 1-2 and mature adults, respectively. Significant differences between insect and mammalian systems exist with respect to ferritin/transferrin-related iron metabolism (Kosmidis et al., 2011; Meyron-Holtz et al., 2011). Specifically, unlike in mammals, insect transferrins and ferritins have key roles in iron transport, with insect ferritins usually being secreted proteins. In comparative transcriptome studies of CLas-infected ACP and CLso-infected potato psyllid, the importance of iron-chelating functions was suggested based on over-expression of iron- and iron-transport genes, which suggested the exploitation of ACP by CLas for iron nutrition (Fisher et al., 2014; Vyas et al., 2015), Transferrin, on the other hand, is a glycoprotein required for iron transport, is recognized by bacterial outer membrane receptors of transferrin-iron complexes and is also recognized as a virulence determinant for *Wuchereria bancrofti*, causal agent of elephantiasis, which is transmitted by *Aedes aegypti* in a circulative-propagative manner (Magalhaes et al., 2008). It seems likely that this protein functions similarly in the CLas-ACP pathosystem.

**Table 3:** Immune response and transmission pathway-related genes in response to ‘*Candidatus* Liberibacter asiaticus’ (CLas) infection of ACP.

GO Description	GO ID	Assigned Contigs	Up-regulated contigs	UP%	Down-regulated contigs	Down%
Immune regulating signaling pathway	GO: 0002764	617	130	21	18	3
Toll signaling pathway	GO: 0008069	181	9	5	0	0
JAK/STAT pathway	GO: 0007259	88	6	7	0	0
ABC transporter activity	GO: 0043190	69	6	9	2	3
Protease-binding activity	GO: 0002020	258	49	19	5	2
Antimicrobial response	GO: 0019730	244	48	20	10	4
Pathogenesis	GO: 0009405	665	99	15	13	2
Autophagy	GO: 0006914	1174	226	19	31	3
Endocytosis	GO: 0006897	1665	212	13	28	2
Exocytosis	GO: 0006887	798	120	15	2	1

Further, a cathepsin L1-like gene was highly over-expressed in all stages after CLas infection, especially in instar1-2 with up to 1000-fold change. Cysteine proteinases, which contain various proteinases, including cathepsins B (cathB), C, F, H, K, and L etc., are involved in multiple functions such as extracellular matrix turnover, antigen presentation, processing events, digestion, immune invasion (Verma et al., 2016). Insect cysteine proteinases play roles in tissue remodeling, molting and metamorphosis during development (Wang et al., 2010). In aphid, gut cathepsin indirectly modulates virus transmission. Host plants indirectly influence plant virus transmission by altering gut cysteine protease activity of aphid vectors. The increased activity of cathB and other cysteine proteases at the cell membrane indirectly decreases virus transmission by aphids, indicating that cathB clearly has important functions in the aphid gut. A similar process may occur in ACP in response to CLas infection, and indeed, has been implicated in a previous comparative transcriptome study (Vyas et al., 2015).

Taken together, these results indicated that CLas infection significantly affects immune response-related genes in different life stages of ACP. These transmission-related genes apparently have different roles based on the differential expression patterns observed among the different ACP life stages, which are expected to harbor a life-stage specific ‘physiological environment’. Consistent with a recent study of the CLas-infected ACP by comparative transcriptome analyses, 499 over-expressed DEGs and 279 under-expressed DEGs were identified that were associated with ubiquitination, immune response, the ribosome, endocytosis, cytoskeleton, and detoxification (i.e. insecticide resistant phenotype). The KEGG analysis revealed that most DEGs were involved in endocytosis and the ribosome (Yu et al., 2020). Integrating these new and archived organ-specific transcriptome data, also showed that the top DGEs from each dataset (bacteriome, midgut, and salivary gland) were sorted by major functional groups including ubiquitination, endocytosis, immunity, and ribosomal-related transcripts (Mann et al., 2022).

In summary, these results have shown that gene expression varies significantly among the different CLas-infected ACP developmental and physiologically distinct stages with respect to responsiveness to CLas-pathogenesis. The infection cycle is expected to initiate with the youngest immature instar, and culminates in the mature adult, which serves as the CLas vector to both the plant and psyllid hosts. Inoculated flush leaves serve as the source of CLas inoculum for ingestion and subsequent infection of young ACP offspring that ingest CLas from citrus leaves on which they feed immediately after hatching. The requirement for early-stage CLas infection of the offspring and for efficient acquisition (to salivary glands) that results in efficient transmission by the adult stage suggests that the barrier to further acquisition may be established by the 4-5^th^ immature instar stage. The different ACP gene expression profiles associated with each immature instar group (1,2 and 3,4) or single instar (3), and the teneral and mature adult psyllids reflect a dynamic infection process, and is consistent with a propagative, circulative mode of ACP-mediated CLas transmission. The nymphal instars 4-5 and teneral adults mounted the most striking responses to CLas infection, based on the greatest number of up or down- regulation genes, during the CLas infection cycle, dramatically more-so than the nymphal instars 1-2, instar 3 and mature, or mature adults. Through this global differential analysis of the transcriptome profiles for the CLas-infected and CLas-free ACP host, the five groups (1,2; 3, 4; teneral adult, mature adult) and/or single immature instar-3, which represent distinct developmental stages, distinct life-stage physiological requirements have been revealed in response to CLas infection. Among them are hallmark genes and biological pathways involved in psyllid development, nutrition, immunity in relation to CLas invasion and systemic infection of the ACP host, consistent with and recognizable in well-studied pathosystems and host-parasite interactions. This new knowledge will go far to enhance the understanding of host-pathogen interactions in the numerous psyllid - ‘Ca. Liberibacter’ pathosystems recently recognized as emergent vector-pathogen complexes in agricultural crops worldwide.

## Conclusion

The central hypothesis of this study is that different ACP life stages influence gene expression that reflect differential responsiveness to CLas infection in a manner that is consistent with the circulative, propagative mode of ACP transmission of the CLas pathogen, unusual in its ability to infect both its psyllid and plant host. These results demonstrate that the early psyllid life-stages are critical targets of CLas invasion, multiplication, circulation, and acquisition or entry into the salivary glands and possibly the oral cavity. The CLas pathogen significantly altered the expression of a diverse repertoire of ACP genes, with the nymphal instars 4-5 and teneral adults in particular, exhibiting the greatest changes in gene expression in response to CLas infection, both in magnitude (fold change in expression) and richness (numbers of responsive genes). These observations further suggest that adults, particularly, the teneral and mature adults have evolved some extent of tolerance to CLas infection and/or that CLas may modulate certain ACP physiological processes to slow the accumulation of detrimental pathogenic effects, outcome(s), of which either or both scenarios may be attributable to long-term host-pathogen co-evolution (Fisher et al., 2014; Vyas et al., 2015). Regardless of mechanism, CLas survival is ensured because ACP-mediated CLas transmission occurs before the psyllid host and vector becomes debilitated by the infection and succumbs to death (Cicero et al., 2017).

Age- and lifespan-related factors are known in other insect vector-pathosystems to be determinants of transmission efficiency. Somewhat notable among known plant insect vector-transmitted fastidious bacterial pathosystems, the ACP immature instars or nymphs, rather than adults, are the chief life stages involved in CLas acquisition, albeit this feature has been commonly associated with the circulative, propagative mode of transmission employed by certain plant virus-insect pathosystems. Gene expression patterns, in particular, that were associated with the instar 4–5 group and teneral (immature) adults, revealed that psyllid defenses are most responsive during the transition period between nymph and adult, thereby potentially diminishing the potential for CLas acquisition by ACP during this developmental window. The developmental stage-associated changes in gene expression that reflect direct response to CLas infection and pathogenesis further supports the observation the dynamics between ACP development and CLas infection, acquisition, and transmission (vector) potential are tightly coordinated.

Some or most DEGs identified here represent potential candidate gene targets for further elucidating CLas-ACP interactions involved in transmission through functional genomics studies, and also for translation to RNAi-mediated strategies for ACP/HLB management. The deployment of RNAi as a component of integrated management approaches for ACP-CLas pathosystem has promise as an alternative or complementary strategy to the intensive chemical control of ACP that embodies the primary management strategy advocated for HLB, although that strategy is costly, unsustainable, and has in general, been ineffective in the long-term particularly, once persistent infection of citrus-growing areas has manifested (Hall, 2013).

This study provides the first the evidence for detailed ACP-stage specific differential expression of immature instars 1,2 compared to 3, and 4-5 and the teneral and mature adult stages, and highlights a number of potential candidates for functional genomic, follow-on validation to better understand host-pathogen mechanisms and processes that drive the success of the ACP-CLas pathosystem and has intriguing applications to other insect vector-borne pathogen complexes.

## Data availability statement

The datasets presented in this study can be found in online repositories. The names of the repository/repositories and accession number(s) can be found in the article/**Supplementary Material**.

## Author contributions

RH, DG and JB contributed to the conception and design of the study. RH conducted the RNA-seq library construction and sequencing. RH, TF, SS and MW performed the data analysis. KP-S provided insect samples. JB and DG contributed with research grant funding application and management. RH wrote the first draft of the manuscript. RH, DG and JB wrote the final version of the manuscript. All authors contributed to the article and approved the submitted version.
